# Sodium Caseinate/Tea Polyphenols Stabilized Lavender Essential Oil Nanoemulsions: Preparation, Characterization, Antibacterial Activity and Potential as Natural Food Preservatives

**DOI:** 10.3390/polym18121526

**Published:** 2026-06-19

**Authors:** Yu Chen, Jiaxin He, Haiting Cai, Yanli Cai, Wei Liao, Adem Gharsallaoui, Kai Yang, Peilong Sun, Ming Cai, Jian Wang

**Affiliations:** 1 College of Food Science and Engineering, Zhejiang University of Technology, Hangzhou 310014, China; 2 College of Chemical Engineering, Zhejiang University of Technology, Hangzhou 310014, China; caihaiting@zjut.edu.cn; 3 Xinjiang Ipaerhan Spice Co., Ltd., Kokdala 835219, China; 4 Givaudan Naturals France, 84140 Avignon, France; 5 CNRS, LAGEPP, UMR 5007, University Claude Bernard Lyon 1, University of Lyon, 69100 Lyon, France

**Keywords:** lavender essential oil, nanoemulsion, sodium caseinate, tea polyphenols, stabilization, antibacterial activity, food preservative

## Abstract

Excessive application of chemical preservatives has raised increasing concerns regarding food safety and human health, prompting the search for safer natural alternatives. Lavender essential oil (LEO), a plant-derived antimicrobial agent, has been considered a promising substitute for synthetic preservatives, but its high volatility and poor water solubility limit its practical application. In this study, LEO nanoemulsions were fabricated via high-pressure homogenization using sodium caseinate (SC) and tea polyphenols (TPs) as composite emulsifiers. The preparation process was optimized using a three-factor, three-level orthogonal design, and the physicochemical properties, storage stability, and antibacterial activity were systematically investigated. The optimal preparation conditions were determined as an SC/TP mass ratio of 2:1, homogenization pressure of 70 MPa, and 7 homogenization cycles. The optimized nanoemulsion exhibited a droplet size of 130–210 nm, zeta potential of −30.89 mV, and encapsulation efficiency of 98.61%, with typical shear-thinning behavior and excellent storage stability. The percentage of free LEO remained below 7.5% within 15 days, indicating high stability, and the release behavior followed a zero-order kinetic model. The prepared nanoemulsion showed significant antibacterial activity against *Staphylococcus aureus* and *Escherichia coli*, with a minimum inhibitory concentration (MIC) of 62.5 μg/mL for both strains. This study confirms that the SC/TP composite interface can effectively stabilize LEO nanoemulsions, providing a theoretical basis for the development of natural and efficient food preservatives.

## 1. Introduction

Excessive use of chemical preservatives can lead to hormonal disorders, mutagenicity, genotoxicity, and various degrees of cytotoxicity and sperm toxicity, posing health risks [1]. On the other hand, essential oils, as secondary metabolites of aromatic plants, possess functions such as antibacterial properties and food preservation [2]. Therefore, they have become ideal alternatives to chemical preservatives. Lavender Essential Oil (LEO) is extracted from lavender plants (originating in the Mediterranean region), and its chemical composition, antibacterial efficacy, and growth environment, climate conditions, lavender varieties, and extraction processes are closely related [3]. This essential oil typically appears as a colorless to pale yellow liquid under normal conditions and is insoluble in water, but it can dissolve well in organic solvents [4]. As a representative antibacterial plant essential oil, lavender essential oil contains components such as linalool and linalyl acetate, which have a significant inhibitory effect on foodborne harmful bacteria. It is widely used as a natural plant additive in the food industry.

Despite its strong characteristic aroma, LEO can be potentially applied in food systems such as flavored beverages, confectionery, and functional foods where its sensory profile is acceptable or even desirable. In particular, it is considered suitable for application in ready-to-drink beverages, bakery products, dairy desserts, and edible coatings for fresh produce, where it can serve as a natural alternative to conventional chemical preservatives while also contributing desirable aroma characteristics. Nanoemulsion technology provides an effective solution to these problems: by encapsulating LEO in nanoemulsion, its stability and antioxidant properties can be significantly improved. Among them, high-pressure homogenization, due to its efficiency, low cost, and ease of large-scale preparation, has become the mainstream method for embedding essential oils, and its products can be used directly in liquid form or expanded for application after spray drying or freeze drying [5]. The nanoemulsion prepared by the emulsification method can enhance the stability, dispersion, bioavailability, and reactivity of LEO, and enhance the interaction with the cell membrane, promoting intracellular penetration, thereby enhancing biological activity [6]. Existing studies have shown that the use of LEO nanoemulsion in food can extend the shelf life and enhance antibacterial properties, for example, the LEO nanoemulsion prepared by emulsification with Tween20 has good storage stability, and its antibacterial activity increases with the increase in essential oil concentration [7]. However, the selection of emulsifiers in the preparation process is crucial for nanoemulsion. The safety controversy of synthetic emulsifiers limits their application in natural food additives, and it is urgent to develop safer natural emulsification systems. Sodium caseinate (SC), as a natural high-molecular-weight emulsifier, can form a dense protective film on the surface of LEO droplets due to its abundant hydrophilic and hydrophobic groups in the molecule; Huck-Iriart and his colleagues confirmed that sucrose and sodium caseinate can improve the stability of the emulsion [8]. However, this system has a single function and lacks antioxidants and other synergistic functions. Tea polyphenols (TPs), as natural polyphenolic substances, can form hydrogen bonds and hydrophobic interactions with sodium caseinate to construct a composite emulsification layer, effectively intercepting the diffusion of LEO molecules, theoretically compensating for the defects of a single sodium caseinate. Previous studies have demonstrated that protein–polyphenol interactions can significantly modify interfacial properties and improve emulsion stability, particularly through the formation of covalent and non-covalent complexes between proteins and phenolic compounds [9]. However, current systematic research on this compound system is still scarce, especially lacking in in-depth exploration of factors such as emulsifier ratio, shear pressure, and cycle number in multiple preparation conditions.

Therefore, this study aims to systematically investigate the effects of different preparation conditions on the physicochemical properties, stability, release patterns and antibacterial efficacy of lavender essential oil nanoemulsions. The significance lies in supplementing the theory of essential oil nanoemulsion preparation, revealing the structure-activity relationship and providing a reference for similar systems, as well as providing a basis for the development of natural antibacterial preparations, promoting their application in various fields and solving the problem of microbial contamination. The study will provide support for the application of lavender essential oil in the food industry from multiple dimensions of condition optimization, dynamic tracking of stability, comprehensive fitting of release models and targeted antibacterial effect research.

## 2. Materials and Methods

### 2.1. Materials

The lavender essential oil was obtained from Xinjiang Ipaerhan Spice Co., Ltd. (Kekedala, China), sodium caseinate (molecular weight range: 375–759 kDa) was obtained from Hubei Baiteiwei Biotechnology Co., Ltd. (Xiantao, China), tea polyphenols (EGCG > 90%) were bought from Shanghai Kecheng Biotechnology Co., Ltd. (Shanghai, China), medium-chain triglycerides (MCTs), mainly composed of caprylic and capric triglycerides, were purchased from Liaoning Xinxing Pharmaceutical Co., Ltd. (Tieling, China), anhydrous ethanol was obtained from Zhongshan Huasida Chemical Co., Ltd. (Zhongshan, China), n-hexane was purchased from Tianjin Beike Chemical Co., Ltd. (Tianjin, China), Luria–Bertani broth and LB agar were obtained from Guangdong Huan Kai Biotechnology Co., Ltd. (Guangzhou, China), and *Escherichia coli* and *Staphylococcus aureus* were obtained from laboratory preservation.

### 2.2. Chemical Analyses of LEO in Nanoemulsions

To investigate the volatile compound profile of LEO and its stability in the nanoemulsion formulations, an Agilent 7890B gas chromatograph coupled with an Agilent 5977B mass spectrometer (GC-MS) was employed (Agilent, Santa Clara, CA, USA). LEO in the nanoemulsion was extracted with n-hexane by ultrasonication (30 min, 200 W, Ice bath temperature control), then analyzed by GC-MS. The extraction process was conducted in an ice bath to ensure that no overheating occurred, thereby minimizing potential changes in the volatile components. Separation was carried out using an HP-5MS capillary column (30 m × 0.25 mm, 0.25 μm film thickness). The GC temperature program was as follows: initial temperature 40 °C, held for 2 min, then ramped to 250 °C at 5 °C min^−1^, and finally held for 5 min. Helium was used as the carrier gas at a constant flow rate of 1.0 mL min^−1^. The mass spectrometer was operated in electron impact (EI) mode at 70 eV, with a scan range of 40–500 *m*/*z*. The ion source and transfer line temperatures were set at 230 °C and 280 °C, respectively. The volatile compound profile was identified by comparing their mass spectra with those in the NIST 20 and Wiley 11 mass spectral libraries. Relative quantitative analysis was performed based on peak area percentages from the total ion chromatogram (TIC) without correction factors. All analyses were conducted in triplicate to ensure reproducibility.

### 2.3. Preparation of Sodium Caseinate/Tea Polyphenols Mixture

SC and TP powders were dispersed in deionized water at mass ratios of 1:2, 1:1, and 2:1 to prepare 2% (*w*/*v*) dispersions. This dispersion served as the aqueous phase of the emulsion. The mixtures were magnetically stirred for 2 h at room temperature to ensure complete dispersion, then used within 24 h. Unused dispersions were sealed and stored at 4 °C for up to 3 days to prevent TP oxidation.

### 2.4. Preparation of LEO Nanoemulsions and Its Optimization

The nanoemulsion was prepared using the SC/TP dispersion as the emulsifier. The LEO: MCT and Oil: Emulsifier ratios are established by researchers [10]. The oil phase, a 1:1 (*v*/*v*) mixture of LEO and MCT, was magnetically stirred overnight at 25 °C and stored at 4 °C. The emulsifier and oil phase were mixed at a 95:5 ratio, then high-shear homogenized (HFJ-25, Tianjin HengAo Technology Development Co., Ltd., Tianjin, China) at 12,000 rpm for 2 min to form a coarse emulsion, which was immediately processed in a high-pressure homogenizer (AH-1500, Su Zhou AntuosNano technology Co., Ltd., Suzhou, China) at 30, 50, or 70 MPa for 3, 5, or 7 cycles. Each formulation was prepared in triplicate to ensure reproducibility and to evaluate experimental variability. To investigate the influence of key preparation parameters on the LEO nanoemulsion, an orthogonal experimental design was employed (Table 1). This approach reduces the number of experiments while providing comprehensive information on factor effects, making it a common and efficient method for optimizing colloidal systems. The factors and their levels were set as follows: the mass ratio of SC to TPs (1:2, 1:1, 2:1), homogenization pressure (30, 50, 70 MPa), and homogenization cycles (3, 5, 7). All groups were prepared under strictly controlled conditions: with the preparation temperature maintained at 25 ± 1 °C, and the short processing time minimized excessive temperature increase. The oil phase added to the aqueous phase first for emulsification to eliminate interference from non-target factors and ensure accuracy and repeatability.

Based on the orthogonal experimental results, the optimal preparation conditions were determined as an SC/TP mass ratio of 2:1, a homogenization pressure of 70 MPa, and 7 homogenization cycles. Unless otherwise specified, the nanoemulsion prepared under these optimized conditions was used for all subsequent characterization and functional analyses.

### 2.5. Droplet Size and Zeta Potential

The particle size distribution and zeta potential of LEO nanoemulsions (SC/TP mass ratios 1:2, 1:1, 2:1) were measured at 25 °C using a Zetasizer Nano ZS90 (Malvern Panalytical, Malvern, UK) with dynamic light scattering (DLS). Samples were diluted 1000-fold in ultrapure water to avoid multiple scattering effects, then measured in triplicate. The same diluted samples were used for zeta potential analysis.

### 2.6. Rheological Behavior of LEO Nanoemulsions

The microrheological behavior of the nanoemulsions was measured by a MCR302 modular rotary rheometer (Anton Paar GmbH, Graz, Austria). Briefly, 3 mL of freshly prepared nanoemulsion was evenly spread onto the center of the steel parallel plate (20 mm diameter, 1 mm gap) of the rheometer. It must be ensured that the plate is completely covered by the sample without any air bubbles. The instrument was then started, and a logarithmic shear rate scan was performed from 0.1 s^−1^ to 100 s^−1^. The apparent viscosity (MPa·s) and shear stress (Pa) of the emulsion were recorded in real-time as functions of the shear rate.

### 2.7. Release Kinetics of LEO

The release behavior of LEO in the nanoemulsion was evaluated by the aqueous phase of the nanoemulsion system using an ethanol/n-hexane extraction method. The sample was stored in a refrigerator at 4 °C, and at days 1, 3, 5, 7, 9, 11, 13, and 15, a 1 mL aliquot was withdrawn and transferred into a 50 mL centrifuge tube. Subsequently, 2 mL of anhydrous ethanol and 3 mL of n-hexane were added (nanoemulsion/ethanol/n-hexane, volume ratio 1:2:3). The mixture was thoroughly shaken, and the absorbance of the resulting dispersion was measured at 255 nm using a UV spectrophotometer. The concentration of free LEO was determined from the standard curve, and the cumulative release percentage was calculated using the following formula. Each sample was measured in triplicate, and the average value was reported:

Release percentage (%) = Free LEO (g)/Total LEO (g) × 100(1)

After measuring the content of free LEO, the encapsulation rate of LEO nanoemulsion was calculated, which was used to characterize its sustained-release performance. Then, it was substituted into the conventional release equation for data fitting. After comparison, the optimal essential oil release equation was obtained.

### 2.8. Confocal Laser Scanning Microscope (CLSM)

Microstructural analysis of the nanoemulsions was performed using a Leica TCS SP8 confocal laser scanning microscope (CLSM) (Leica, Wetzlar, Germany). The nanoemulsion used for CLSM observation was prepared under the optimal conditions determined by orthogonal analysis, namely an SC/TP mass ratio of 2:1, a homogenization pressure of 70 MPa, and 7 homogenization cycles. Nile Red (for labeling LEO) and FITC (for labeling TP) were prepared as 0.01% (*w*/*v*) dispersions in ethanol (≥95%). For staining, 1 mL of nanoemulsion was mixed with 20 μL each of Nile Red and FITC dispersions, vortexed, and incubated in the dark at room temperature for 20 min. A 10 μL aliquot was then placed on a glass slide, covered with a coverslip (avoiding bubbles), and excess liquid was removed.

### 2.9. Encapsulation Efficiency (EE) of LEO Nanoemulsions

The encapsulation efficiency (EE) of LEO in the nanoemulsions was determined using a GENESYS 150 UV-Vis spectrophotometer (Thermo Fisher Scientific, Waltham, MA, USA). Anhydrous ethanol, n-hexane, and the freshly prepared LEO nanoemulsion were mixed in a volume ratio of 2:3:1 in a 50 mL centrifuge tube. After sealing, the mixture was thoroughly shaken manually. The absorbance of the supernatant was measured at 255 nm. The concentration of free LEO in the samples was calculated from the standard curve, and the EE was determined using the following formula:

EE (%) = (Total LEO (g) − Free LEO (g))/Total LEO (g) × 100(2)

### 2.10. Antibacterial Activity of LEO Nanoemulsions

#### 2.10.1. Inhibition Zone of LEO Nanoemulsions

The antibacterial activity of the nanoemulsion was evaluated using the inhibition zone method. LB nutrient agar plates were prepared, and bacterial suspensions of *Escherichia coli* and *Staphylococcus aureus* were provided by the Laboratory of Zhejiang University of Technology (Hangzhou, China). The bacterial suspensions were prepared and were adjusted to approximately 10^7^ CFU/mL. The bacterial suspension was uniformly spread onto the agar surface, and an Oxford cup was placed on top. A 50 μL aliquot of the nanoemulsion sample was then added into the cup. The plates were placed in a 4 °C refrigerator for 60 min to allow for diffusion and subsequently transferred to a 37 °C incubator for inverted incubation for 18–24 h. The antibacterial activity of the emulsion was evaluated by measuring the diameter of the resulting inhibition zone.

#### 2.10.2. The Minimum Inhibition Concentration (MIC) of LEO Nanoemulsions

The MIC value was determined by the broth dilution method with slight modifications. Briefly, the freshly prepared emulsion (at the optimal ratio) was mixed with nutrient broth and serially diluted two-fold to achieve final LEO concentrations of 2000, 1000, 500, 250, 125, 62.5, 31.25, and 15.625 μg/mL. A 20 μL aliquot of the bacterial suspension (approximately 10^7^ CFU/mL) was then added to each tube. Positive controls consisted of nutrient broth mixed with the bacterial suspension, while sterile negative controls contained nutrient broth mixed with the optimal LEO nanoemulsion. After 24 h of incubation, the MIC was defined as the lowest concentration in the dilution series that showed no visible microbial growth.

### 2.11. Statistical Analysis

All measurements were conducted at least three times, and the values were recorded as the mean ± standard deviation (SD). One-way analysis of variance (ANOVA) and the Tukey test in the Statistix8.1 software package were used to compare the means. The comprehensive score for each formulation was calculated as the arithmetic mean of the three normalized scores, assigning equal weight to each indicator. ANOVA was performed using the mean values of the orthogonal experimental runs. A *p* value < 0.05 was considered statistically significant.

## 3. Results and Discussion

### 3.1. Characterization of Nine LEO Nanoemulsion Formulations

**Figure 1:**
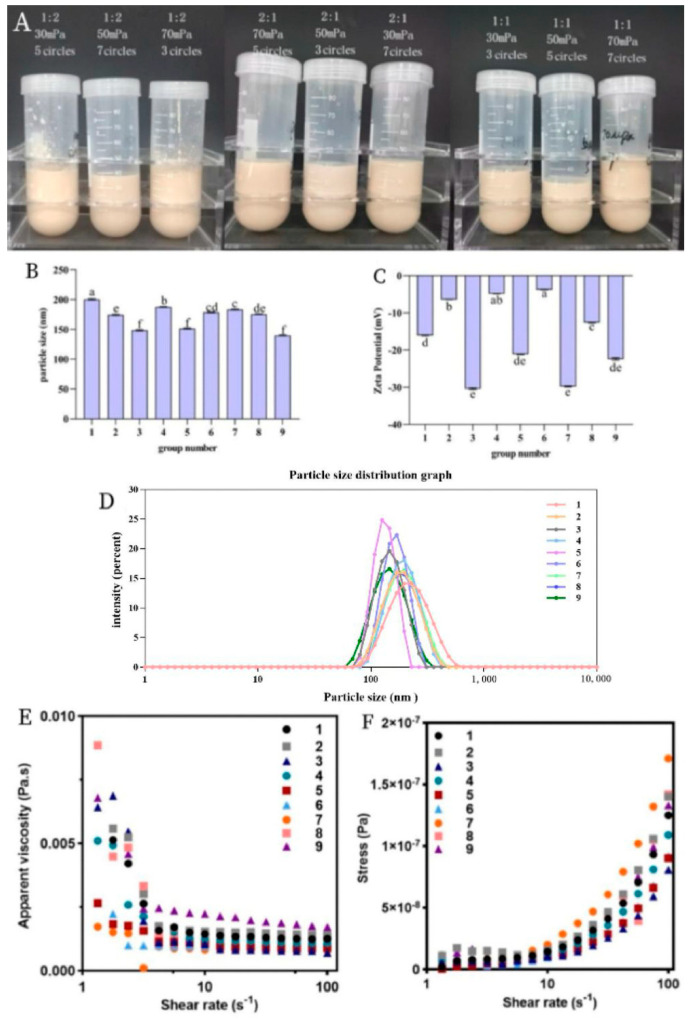
Comprehensive characterization of LEO nanoemulsions prepared under different orthogonal experimental conditions shown in Table 1: (**A**) Appearance of freshly prepared nanoemulsions (from left to right, the corresponding numbers are 4, 5, 6, 9, 8, 7, 1, 2, and 3); (**B**) Droplet size; (**C**) Zeta potential; Different lowercase letters above the bars indicate significant differences among the groups (*p* < 0.05). Bars sharing the same lowercase letters are not significantly different. (**D**) Particle size distribution; (**E**) Apparent viscosity as a function of shear rate; (**F**) Shear stress as a function of shear rate.

A comprehensive characterization was performed on the nine prepared LEO nanoemulsion formulations to evaluate their physicochemical properties, including particle size, PDI, zeta potential, and morphology, thereby providing a systematic basis for assessing the impact of different preparation parameters on emulsion stability and performance. All results presented in Figure 1 should be interpreted in conjunction with the orthogonal design shown in Table 1. The complete particle size, PDI, and zeta potential data for all nine formulations are provided in Tables S1 and S4. All measurements were performed in triplicate and are presented as mean ± standard deviation (SD).

#### 3.1.1. Appearance of LEO Nanoemulsions

Nine groups of LEO nanoemulsions were prepared according to the scheme (Table 1). As shown in Figure 1A, all fresh emulsions exhibited no obvious stratification, precipitation, or flocculation, indicating good initial stability. This suggests that, within the tested factor levels, the composite emulsifying system combined with high-pressure homogenization effectively maintains emulsion stability, consistent with the findings of Huck-Iriart et al. [8]. Their study reported that SC-based emulsifiers stabilize the interfacial film to inhibit oil droplet aggregation. The current study further confirms that introducing TPs not only does not weaken this stability but may enhance it through intermolecular interactions. Furthermore, the emulsion color changed from light yellow to light brown as the SC:TP ratio increased from 1:2 to 2:1. This trend is attributed to TP: (1) TP is susceptible to oxidative polymerization during the preparation process, generating brown pigments that cause the emulsion color to darken; (2) at higher SC:TP ratios (e.g., 2:1), stronger hydrogen bonds and hydrophobic interactions between SC and TPs facilitate the formation of complex polymer aggregates, which increases the system turbidity and alters light propagation, thus further intensifying the color deepening. This mechanism is consistent with the conclusions of researchers, who elucidated the effect of polyphenol oxidation on the color variation of protein–polyphenol composite stabilized emulsions [11].

#### 3.1.2. Particle Size and Zeta Potential of Nanoemulsions

Analysis of the data presented in Figure 1B,D (corresponding to formulations No. 1–9 in Table 1) revealed that the droplet diameters of all LEO nanoemulsions prepared in this study ranged from 130 to 210 nm, consistent with the typical characteristics of nanoemulsions (droplet diameters usually <200 nm). To facilitate interpretation, Table 1 explicitly lists the composition and processing parameters corresponding to each nanoemulsion number. This finding confirms the successful formation of a stable nanoscale dispersion system. A decreasing trend in droplet size was observed with increasing SC proportion, homogenization pressure, and homogenization cycles. However, due to the orthogonal experimental design, these trends represent the combined effects of multiple factors rather than the independent influence of each parameter. To facilitate comparison among formulations and improve data transparency, the complete particle size, PDI, and zeta potential data for all nine formulations are presented in Table S1 as mean ± SD values (*n* = 3). Because the orthogonal design simultaneously varies multiple factors, these data are presented primarily to provide transparency and allow future analyses, rather than to establish independent single-factor effects. The observed reduction in droplet size under certain formulation conditions may be associated with increasing SC proportion. This is attributed to the adsorption of more emulsifier molecules at the LEO droplet interface, forming a dense protective film that inhibits droplet aggregation and coalescence [12]. Higher homogenization pressure and additional homogenization cycles are generally expected to increase mechanical shear forces and facilitate droplet disruption [13]. As shown in Figure 1C, concurrent with the changes in particle size, the absolute value of the zeta potential of the LEO nanoemulsions increased with the SC proportion. Zeta potential is a key indicator of particle surface charge; higher absolute values indicate greater surface charge density and stronger electrostatic repulsion. In aqueous dispersions, SC molecules carry a net negative charge due to the ionization of carboxyl groups along the polypeptide chain. Increasing the SC proportion results in the adsorption of more negative charges on the LEO droplet surfaces, thereby increasing the absolute zeta potential value. This enhanced electrostatic repulsion prevents particles from approaching each other and aggregating, thereby significantly improving the stability of the system.

#### 3.1.3. Rheological Behavior Analysis

Analysis of the rheological data presented in Figure 1E,F indicates that all LEO nanoemulsions exhibited typical shear-thinning behavior as the shear rate increased from 1 to 100 s^−1^ [14]. With increasing shear rate, the apparent viscosity of the emulsions decreased, while the shear stress increased linearly, reflecting dynamic adjustments in the microstructure of the emulsion system under shear [15]. As shown in Figure 1E, the degree of shear thinning varied with the emulsifier mass ratio; however, due to the orthogonal experimental design, this observation reflects the combined influence of multiple processing parameters rather than the effect of any single factor. To improve clarity, representative viscosity values at selected shear rates are summarized in Table S2. In addition, the influence of each factor was evaluated based on average data at different levels, rather than relying solely on visual comparison of the curves. At an SC:TP mass ratio of 1:2, the emulsion exhibited the lowest apparent viscosity, suggesting that the internal structure was more susceptible to disruption by shear forces, resulting in a more pronounced decrease in viscosity. Conversely, at a mass ratio of 2:1, the apparent viscosity was higher. This is attributed to the formation of a denser interfacial layer at this ratio, which enhances interactions between oil droplets and thereby increases the apparent viscosity of the system.

#### 3.1.4. Stability of LEO Nanoemulsions

**Figure 2:**
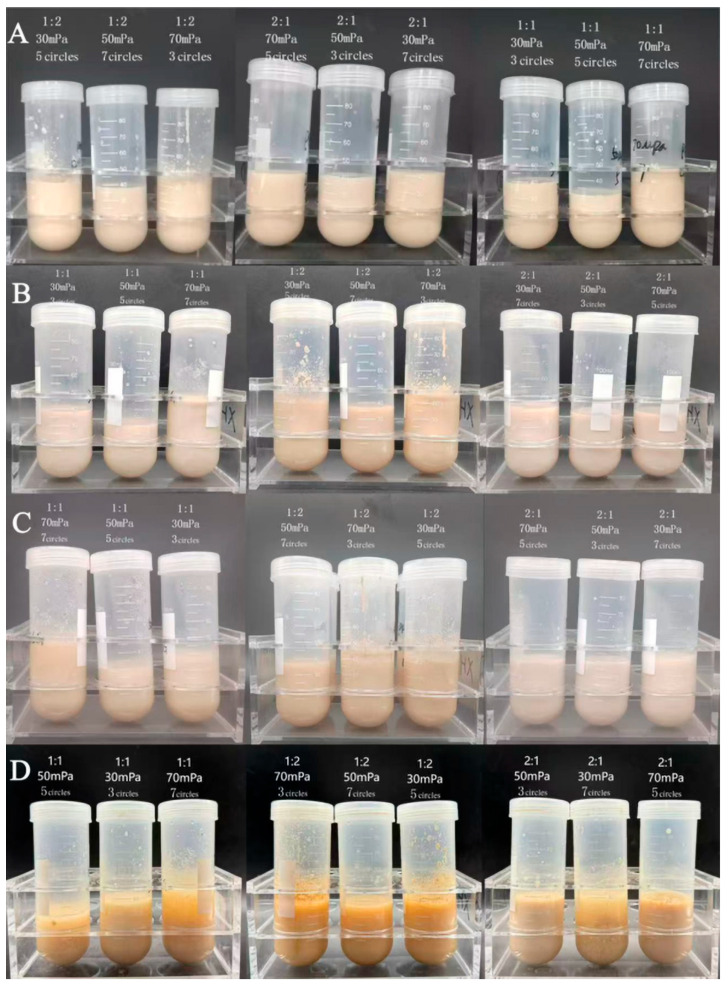
Storage stability of LEO nanoemulsions at 4 °C for 20 days: (**A**) Day 1, (**B**) Day 7, (**C**) Day 10, (**D**) Day 20.

As shown in Figure 2, the mass ratio of SC to TPs significantly influenced the physical stability of the emulsions. At a 1:2 ratio, the emulsion exhibited the poorest stability, with slight precipitation observed on day 7 and obvious stratification by day 10. This instability is attributed to the relatively low concentration of SC, which is insufficient to form a complete and continuous interfacial film on the oil droplet surfaces. Additionally, the excessive amount of TPs may disrupt the charge balance of the system, thereby intensifying particle aggregation. At a 1:1 ratio, obvious stratification and flocculation occurred by day 20. Although the emulsifier concentration at this ratio is moderate, the mechanical strength of the interfacial film may be inadequate. Upon prolonged storage, the electrostatic repulsion between oil droplets weakens, leading to aggregation and sedimentation. The highest stability was observed at a 2:1 ratio, with only slight stratification noted on day 20 in the sample homogenized at 30 MPa. At this higher SC concentration, a dense interfacial film is formed through hydrophobic interactions, and the phenolic hydroxyl groups of TPs are integrated into this film via hydrogen bonding. This cooperative effect enhances spatial steric hindrance, thereby effectively inhibiting oil droplet aggregation [16]. Similarly, Liu et al. reported that cinnamon essential oil nanoemulsions stabilized with OSA-modified starch also exhibited good storage stability [17].

#### 3.1.5. Analysis of Orthogonal Test Results of LEO Nanoemulsions

**Figure 3:**
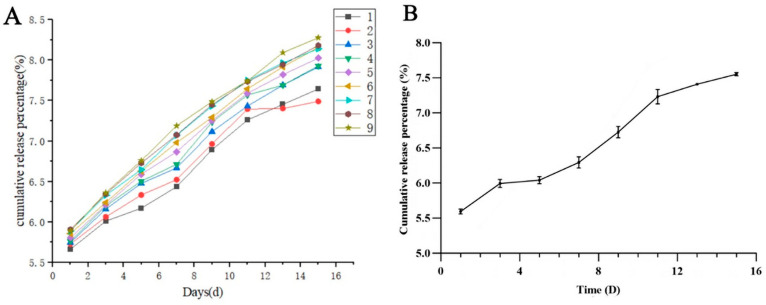
Cumulative release rate of LEO from nanoemulsions prepared under different conditions (**A**) and the optimized LEO nanoemulsion (**B**) during 15-day storage at 4 °C.

Due to the orthogonal experimental design, the trends observed in Figure 3 reflect the combined effects of multiple factors, although single-factor analysis can still provide useful insights into the influence of individual variables on specific parameters. To achieve a more comprehensive evaluation of emulsion performance, a multi-index comprehensive scoring method based on orthogonal analysis was applied. The normalized orthogonal test results of LEO nanoemulsion are shown in Table 2. In this method, key indicators including droplet size, PDI, and zeta potential were first normalized, and then weighed equally to calculate a comprehensive score for each experimental group. The comprehensive score was obtained by summing the normalized values of each parameter, allowing for an overall comparison of emulsion performance. Analysis of variance based on the comprehensive score indicated that the tested factors contributed differently to the overall performance of the nanoemulsions. However, none of the factors reached statistical significance at the 95% confidence level (*p* > 0.05). Among them, the SC:TP ratios (A), homogenization pressure (B), and homogenization cycles (C) all have varying degrees of impact on emulsion performance. Column D represents a dummy factor introduced in the orthogonal design for error estimation and does not correspond to an actual experimental variable. Based on the orthogonal comprehensive scoring method, although none of the investigated factors reached statistical significance at the 95% confidence level (*p* > 0.05), range analysis revealed differences in their relative contributions to the comprehensive score. The magnitude of factor influence was assessed using the R value (range of level means), which ranked the factors as homogenization pressure (B) > SC:TP ratio (A) > homogenization cycles (C). These results should be interpreted as an indication of relative influence within the tested experimental space rather than as evidence of statistically significant differences. Because the comprehensive score integrates droplet size, PDI, and zeta potential simultaneously, Table 2 reflects the overall performance of the nanoemulsions rather than the isolated influence of individual factors on specific physicochemical properties. Therefore, the orthogonal analysis was used primarily to identify the optimal preparation conditions rather than to quantify the independent effect of each factor. Based on the comparison of comprehensive scores under different levels of each factor, the optimal combination was determined to be A_3_B_3_C_3_, namely a SC:TP ratios of 2:1, a homogenization pressure of 70 MPa, and 7 homogenization cycles. Within the scope of this experiment, homogenization pressure is the key factor affecting the performance of LEO nanoemulsions, followed by homogenization cycles, while the SC:TP ratios have a relatively small effect. The prepared LEO nanoemulsion which using the optimal scheme had a particle size of 142.8 nm, a PDI of 0.0899, and a Zeta potential of −30.89 mV, indicating a uniform particle size distribution and good system stability.

### 3.2. Encapsulation Efficiency (EE) of LEO Nanoemulsion

The encapsulation efficiency (EE) of the freshly prepared LEO nanoemulsion was determined, yielding a value of 98.61%. This high EE indicates that under the optimized conditions (SC:TP mass ratio of 2:1, homogenization pressure of 70 MPa, and 7 cycles), the encapsulation process was highly effective. The dense interfacial emulsifier membrane formed under these conditions acts as a physical barrier, preventing direct contact between the encapsulated LEO and the external environment. Furthermore, the stable nano-dispersed system reduces the diffusion rate of LEO molecules, thereby jointly delaying their volatilization [18].

### 3.3. Chemical Composition Analysis of Lavender Essential Oil

The chemical compositions of fresh and stored lavender essential oils are summarized in Table 3. After excluding contaminants, column-related artifacts, and compounds not recognized as authentic constituents of lavender essential oil, 25 compounds were retained for analysis. The identified constituents mainly belonged to monoterpene hydrocarbons, oxygenated monoterpenes, esters, and sesquiterpene derivatives. The fresh essential oil was characterized by a predominance of oxygenated monoterpenes and esters, with linalool (41.0%) and linalyl acetate (35.2%) representing the major constituents, followed by β-myrcene (7.1%), terpinen-4-ol (7.6%), geranyl acetate (6.6%), trans-β-ocimene (4.2%), and (Z)-β-ocimene (3.3%). Linalool and linalyl acetate are widely recognized as key quality markers of lavender essential oil because they contribute to the characteristic floral, sweet, and fresh aroma associated with premium lavender products [19]. The predominance of these compounds, together with the relatively low abundance of camphor, suggests that the investigated oil possessed the compositional characteristics generally associated with high-quality lavender essential oil. Similar constituent profiles have also been reported linalool and linalyl acetate as the dominant constituents of lavender essential oil analyzed by GC-MS [20]. The relatively high abundance of oxygenated monoterpenes and esters observed in the present study further supports the authenticity and quality of the investigated oil.

Storage induced noticeable changes in the relative abundance of several constituents. Monoterpene hydrocarbons and some oxygenated monoterpenes generally decreased during storage, including β-myrcene, trans-β-ocimene, (Z)-β-ocimene, terpinen-4-ol, and linalool. These compounds possess relatively high volatility and are particularly susceptible to evaporation and oxidative degradation. Linalool decreased from 41.0% to 37.3%, β-myrcene decreased from 7.1% to 4.0%, terpinen-4-ol decreased from 7.6% to 2.9%, and (Z)-β-ocimene decreased from 3.3% to 1.5%, while trans-β-ocimene was no longer detected after storage. Linalyl acetate increased from 35.2% to 42.0%, and geranyl acetate increased from 6.6% to 7.4%. This apparent increase most likely reflects the preferential loss of highly volatile compounds, resulting in a relative enrichment of less volatile ester constituents. Trans-linalool oxide (furanoid) and caryophyllene oxide also increased markedly during storage, from 0.5% to 2.1% and from 0.2% to 1.9%, respectively. Oxygenated derivatives tended to increase during storage and processing of lavender essential oils, suggesting ongoing oxidation of terpene precursors [21]. The observed decrease in monoterpene hydrocarbons together with the increase in oxygenated derivatives indicates that volatilization and oxidation were the major processes governing compositional changes during storage.

Despite these compositional changes, linalool and linalyl acetate remained the predominant constituents after storage, indicating that the characteristic composition of lavender essential oil was largely maintained. From an aroma perspective, reductions in ocimenes and myrcene may weaken fresh green and herbaceous notes, whereas the relative enrichment of ester compounds may enhance sweet and floral characteristics. The formation of oxygenated derivatives such as linalool oxides and caryophyllene oxide further reflects gradual oxidative aging of the oil. Nevertheless, the retention of the major aroma-active constituents demonstrates that the essential oil maintained good compositional stability throughout storage. Similar storage-related compositional shifts have been reported among commercial lavender oils originating from different sources, highlighting the importance of storage conditions and quality control for maintaining product authenticity [22]. Overall, storage primarily affected minor and medium-abundance volatile constituents, while the compounds most responsible for lavender aroma quality remained predominant.

### 3.4. CLSM Analysis

**Figure 4:**
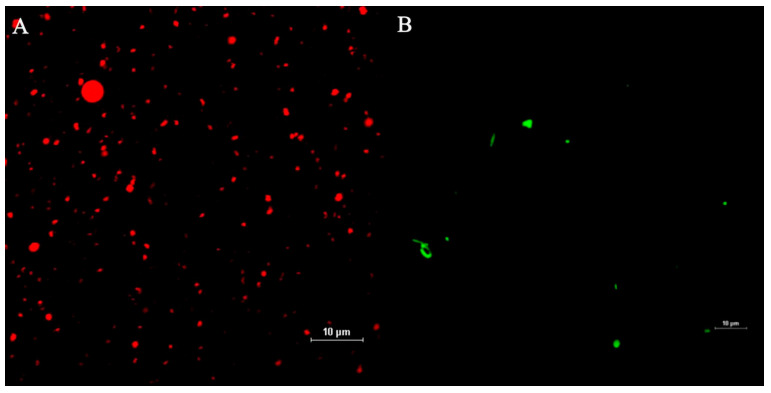
CLSM micrographs of optimized LEO nanoemulsion: (**A**) Nile Red-stained oil phase (LEO), and (**B**) FITC-stained TPs in the composite emulsifier.

As illustrated in the CLSM images in Figure 4, after specific staining, the components of the LEO nanoemulsion exhibited distinct spatial distributions under microscopic observation. Nile red, a lipophilic dye, preferentially partitions into the oil core, rendering the LEO as red fluorescent signals. The green, fluorescent signals represent labeled TPs, whose phenolic hydroxyl groups can form covalent bonds with the staining agent (FITC), enabling visualization. These images confirm that TP molecules interact synergistically with SC through hydrogen bonding, hydrophobic interactions, and electrostatic adsorption, forming a protective layer at the oil–water interface. This interfacial network effectively inhibits oil droplet aggregation and coalescence, thereby maintaining the stable dispersion of the LEO nanoemulsion at the microscopic level. This observation is consistent with previous findings on emulsions stabilized by polyphenol–chitosan complexes [23].

### 3.5. LEO Emulsion Stability

**Figure 5:**
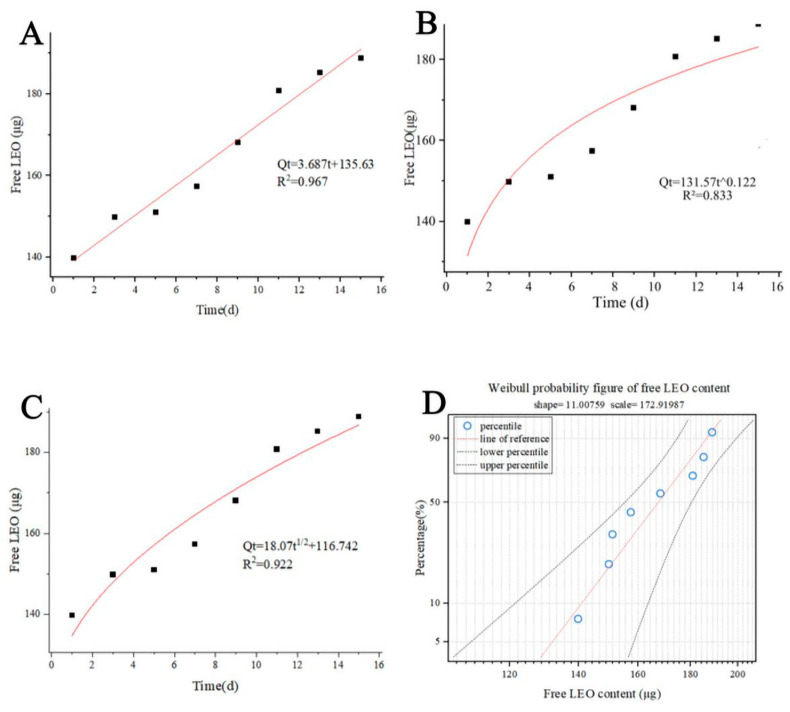
Kinetic model fitting of LEO release from the optimized nanoemulsion: (**A**) Zero-order, (**B**) First-order, (**C**) Higuchi, and (**D**) Weibull model. Black squares represent the experimental release data, and the red lines represent the fitted kinetic model curves. In the Weibull probability plot (**D**), blue circles represent the experimental percentile data, while the gray lines indicate the reference line and the upper and lower percentile limits.

The LEO emulsion stability in Figure 5A shows that the LEO nanoemulsion prepared at an SC:TP mass ratio of 2:1, a homogenization pressure of 70 MPa, and 7 homogenization cycles exhibited distinct release behavior and superior stability over a 15-day storage period. In addition, the droplet size and zeta potential of the optimized nanoemulsion were monitored during storage and showed no significant changes. Meanwhile, the cumulative release percentage remained below 7.5% throughout the storage period, further confirming the excellent physical stability of the system over time. The cumulative release percentage of this sample remained below 7.5% throughout the storage period, which was lower than that of emulsions prepared under other processing conditions. Furthermore, the release curve showed a highly gradual increase after the 12th day, confirming the excellent stability of this formulation. Figure 5B presents the free LEO of the same nanoemulsion refrigerated at 4 °C. The low release rate observed under refrigerated storage indicates that the LEO nanoemulsion maintains good stability under these conditions, which can effectively extend its shelf life. This observation is consistent with the findings reported by Flekka et al. [24].

As shown in the fitting results presented in Figure 5, among several release kinetic models including the zero-order model, first-order kinetic model, Higuchi model, and Weibull kinetic model, the zero-order kinetic model yielded the highest coefficient of determination (R^2^ = 0.97), which was significantly higher than those of the other models. This indicates that the zero-order kinetic model provided the best fit for the essential oil release data from the LEO nanoemulsions and most accurately described the linear relationship between the cumulative release amount of essential oil and time in this system, consistent with the findings reported by Li et al. [25]. The essential oil release equation derived was Qt = 3.687t + 135.63, with a correlation coefficient of R^2^ = 0.97. Considering that the LEO nanoemulsions in this study were stored at 4 °C, the low-temperature environment likely inhibited the intense movement of essential oil molecules [26], resulting in a release process primarily controlled by the properties of the carrier material itself and thus exhibiting stable linear release characteristics.

### 3.6. Antibacterial Activity of LEO Nanoemulsions

**Figure 6:**
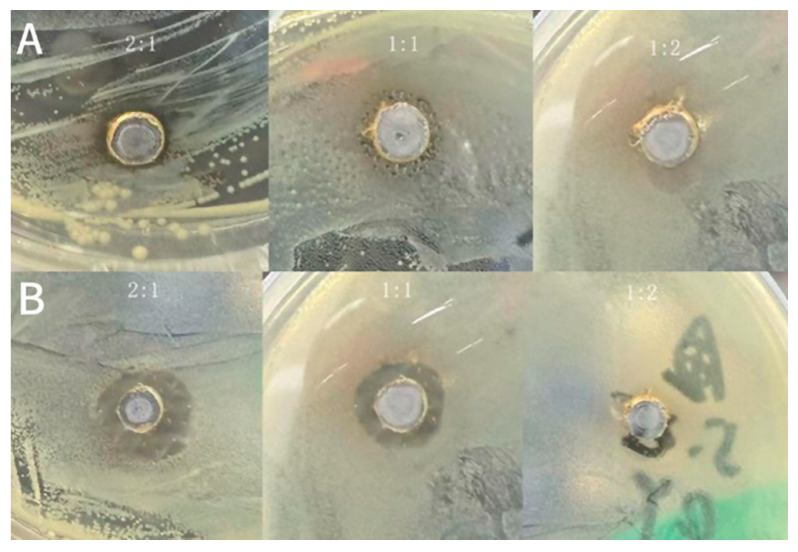
Inhibition zone of LEO nanoemulsions with different SC:TP ratios against *Escherichia coli* (**A**), and *Staphylococcus aureus* (**B**).

As shown in the inhibitory zone images (Figure 6), the nanoemulsion prepared at an SC:TP mass ratio of 2:1 showed the largest antibacterial zone diameter, followed by that at a 1:1 ratio. In contrast, no obvious antibacterial zone was observed for the nanoemulsion prepared at a 1:2 ratio. Additionally, the antibacterial effect of the LEO nanoemulsions against *Staphylococcus aureus* was stronger than that against *Escherichia coli* [27]. This difference in antibacterial activity may be attributed to the inherent structural differences in the cell walls of the two bacterial strains. The cell wall of *Staphylococcus aureus* is primarily composed of a thick peptidoglycan layer and lacks an outer membrane barrier. Hydrophobic active components in LEO (e.g., terpenoids) can directly penetrate the peptidoglycan layer and target the bacterial cell membrane, disrupting the integrity of the lipid bilayer and leading to the leakage of intracellular substances [28]. In contrast, the outer membrane of *Escherichia coli* contains a barrier formed by lipopolysaccharides and lipoproteins, which significantly reduces the penetration efficiency of hydrophobic essential oil components. These components must be slowly transported through outer membrane protein channels, resulting in a relatively delayed antibacterial effect.

**Figure 7:**
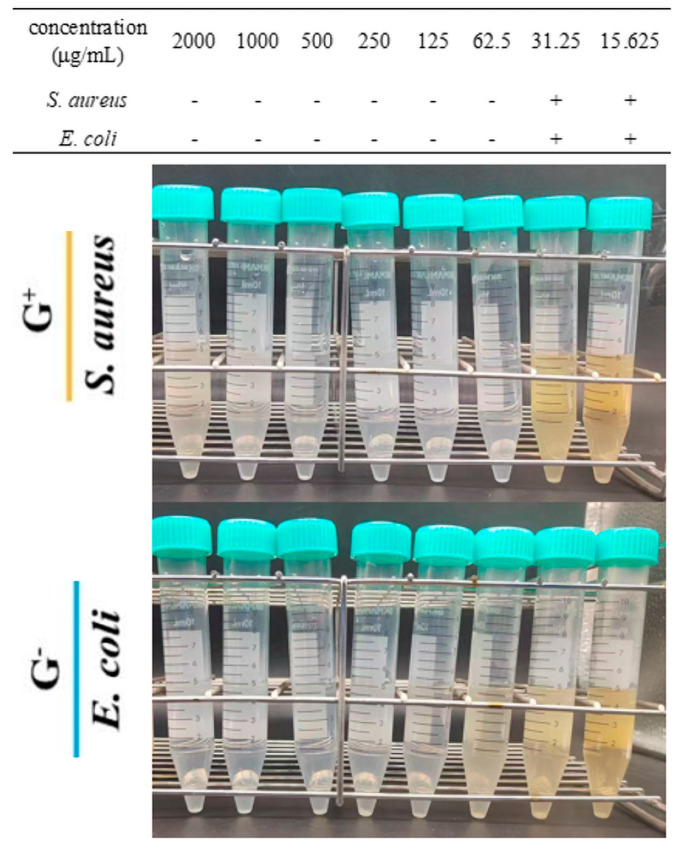
Growth status of *Staphylococcus aureus* and *Escherichia coli* cultured with LEO nanoemulsions at different concentrations. Note: + represents bacterial growth, and − represents bacterial non-growth.

The MIC of the optimized LEO nanoemulsion was further determined (Figure 7), where + represents bacterial growth and − represents non-growth. The results showed that the MIC against both *Staphylococcus aureus* and *Escherichia coli* was 62.5 μg/mL. At concentrations ≥62.5 μg/mL, no visible bacterial growth was observed, indicating complete inhibition; at concentrations <62.5 μg/mL, bacterial growth resumed, confirming the loss of inhibitory effect. Notably, despite the stronger inhibitory zone against *Staphylococcus aureus*, the MIC values for both strains were identical, suggesting that the nanoemulsion system effectively overcomes the outer membrane barrier of *Escherichia coli* to achieve comparable inhibitory efficacy against both pathogens. Compared with conventional bulk essential oils, nanoemulsified systems have been reported to exhibit significantly enhanced antibacterial activity. For instance, nanoemulsions of sage essential oil showed approximately four-fold higher antibacterial activity than the corresponding bulk oil against *E. coli*, despite the oil being dispersed in a diluted form Moghimi et al. [29]. This enhancement is mainly attributed to the reduced droplet size and improved dispersion, which facilitates closer interaction between bioactive compounds and bacterial cell membranes, leading to more effective membrane disruption. Vanegas et al. also reported optimization of the broth dilution method in their study [30].

## 4. Conclusions

This study prepared and optimized LEO-loaded nanoemulsions via high-pressure homogenization with SC/TP as composite emulsifiers, with the optimal parameters being an SC/TP mass ratio of 2:1, 70 MPa homogenization pressure, and 7 cycles. The optimized nanoemulsion had a droplet size of 130–210 nm and a zeta potential of −30.89 mV, forming a stable, uniformly dispersed nanocolloidal system. GC-MS analysis revealed that fresh LEO’s main components were linalool (41.01%) and linalyl acetate (35.17%), while free LEO showed severe volatilization, oxidation, and degradation after storage. CLSM and rheological results confirmed SC and TPs formed a dense composite interfacial film through hydrogen bonding and hydrophobic interactions, endowing the nanoemulsion with typical shear-thinning behavior and excellent mechanical stability. The optimized nanoemulsion had a high EE of 98.61%, with the percentage of free LEO remaining below 7.5% within 15 days and free LEO fitting well with the zero-order kinetic model. Antibacterial assays showed it exerted significant inhibitory effects on *Staphylococcus aureus* and *Escherichia coli* (MIC = 62.5 μg/mL for both), with the mechanism related to LEO active components damaging bacterial cell membrane integrity and inhibiting metabolism. Compared with conventional single-emulsifier systems, the SC/TP composite interface provides synergistic stabilization by combining interfacial structuring and antioxidant protection, effectively improving both the physicochemical stability and functional performance of LEO nanoemulsions. This work not only offers a green and efficient strategy for stabilizing volatile essential oils but also provides new insights into the design of protein–polyphenol composite interfaces for nanoemulsion systems, thereby expanding their potential applications as natural alternatives to chemical preservatives in food systems, like milk tea.

## Figures and Tables

**Table 1:** Orthogonal experimental design for the preparation of LEO nanoemulsions.

Number	Emulsifier Ratio (A)	Homogenizing Pressure (B, MPa)	Homogeneous Cycles (C)
1	1:1	30	3
2	1:1	50	5
3	1:1	70	7
4	1:2	30	5
5	1:2	50	7
6	1:2	70	3
7	2:1	30	7
8	2:1	50	3
9	2:1	70	5

**Table 2:** Analysis table of orthogonal test results of LEO nanoemulsions.

Test Serial Number	Factor				Comprehensive Score
	A (Emulsifier Ratio)	B (Homogeneous Pressure, MPa)	C (Homogenization Cycle)	D	
1	1	1	1	1	42.69
2	1	2	2	2	44.53
3	1	3	3	3	86.54
4	2	1	2	3	29.69
5	2	2	3	1	48.74
6	2	3	1	2	22.39
7	3	1	3	2	75.55
8	3	2	1	3	56.35
9	3	3	2	1	83.83
K1	174.63	190.57	185.03	164.03	
K2	172.63	167.20	167.43	178.90	
K3	166.20	155.70	161.00	170.53	
R	8.43	34.87	24.03	14.87	
Order of priorities	B > C > A				
Excellent level	4	1	2	3	
Factor	Degrees of freedom	Sum of squared deviations	Mean square value	F	Significance
A	2	2253.75	1126.88	10.20	0.09
B	2	430.52	215.26	1.95	0.34
C	2	1346.33	673.17	6.09	0.14
Error	2	221.06	110.53		
Total	8.00	4251.66			

**Table 3:** Relative contents of main volatile components in fresh and stored LEO nanoemulsions.

Compound	CAS	Molecular Formula	Fresh RT (min)	Stored RT (min)			Fresh Area (%)		Stored Area (%)	
α-Phellandrene	99-83-2	C_10_H_16_	6.451	–			0.2		–	
α-Pinene	80-56-8	C_10_H_16_	6.594	6.601			0.3		0.2	
Camphene	79-92-5	C_10_H_16_	6.888	6.894			0.1		0.2	
1-Octen-3-ol	3391-86-4	C_8_H_16_O	7.302	7.308			0.7		0.5	
β-Myrcene	123-35-3	C_10_H_16_	7.468	7.475			7.1		4.0	
m-Cymene	535-77-3	C_10_H_14_	8.102	8.109	0.3	0.4				
Limonene	138-86-3	C_10_H_16_	8.183	8.189	0.9	0.8				
trans-β-Ocimene	3779-61-1	C_10_H_16_	8.229	–	4.2	–				
(Z)-β-Ocimene				3338-55-4	C_10_H_16_		8.409	8.416	3.3	1.5
γ-Terpinene				99-85-4	C_10_H_16_		8.643	–	0.3	–
trans-Linalool oxide (furanoid)				34,995-77-2	C_10_H_18_O_2_		8.853	8.860	0.5	2.1
α-Terpineol				98-55-5	C_10_H_18_O		9.104	10.839	0.4	1.0
Linalool				78-70-6	C_10_H_18_O		9.291	9.291	41.0	37.3
(2E,6Z)-Dimethylocta-2,4,6-triene				7216-56-0	C_10_H_16_		9.724	–	2.4	–
3,4-Dimethylocta-2,4,6-triene				57396-75-5	C_10_H_16_		9.931	–	0.2	–
Camphor				76-22-2	C_10_H_16_O		10.145	10.148	0.3	0.5
Perillyl alcohol				536-59-4	C_10_H_16_O		10.292	10.298	0.5	0.5
Borneol				507-70-0	C_10_H_18_O		10.522	10.529	0.8	0.8
Terpinen-4-ol				562-74-3	C_10_H_18_O		10.629	10.632	7.6	2.9
Geraniol				106-24-1	C_10_H_18_O		11.216	–	0.2	–
Linalyl acetate				115-95-7	C_12_H_20_O_2_		11.553	11.557	35.2	42.0
Geranyl acetate				105-87-3	C_12_H_20_O_2_		12.020	12.024	6.6	7.4
Bornyl acetate				76-49-3	C_12_H_20_O_2_		12.147	12.147	0.1	0.1
Neryl acetate	141-12-8	C_12_H_20_O_2_	13.072	13.075	1.7	1.8				
Caryophyllene oxide	1139-30-6	C_15_H_24_O		16.159	16.155	0.2	1.9			

## Data Availability

The original contributions presented in this study are included in the article/Supplementary Materials. Further inquiries can be directed to the corresponding authors.

## Supplementary Materials

The following supporting information can be downloaded at: https://www.mdpi.com/article/10.3390/polym18121526/s1, Table S1: Univariate Mean Table; Table S2: Apparent Viscosity Data Change; Table S3: Precipitation of LEO nanoemulsions during storage; Table S4: Orthogonal design matrix and ANOVA results for particle size, PDI and zeta potential (ANOVA were based on the orthogonal design shown in Table S1).

## References

[1] Islam F., Saeed F., Imran A., Shehzadi U., Ali R., Nosheen F., Chauhan A., Asghar A., Ojukwu M. (2023). Bio-preservatives and essential oils as an alternative to chemical preservatives in the baking industry: A concurrent review. J. Food Sci. Technol..

[2] Bakkali F., Averbeck S., Averbeck D., Idaomar M. (2008). Biological effects of essential oils–A review. Food Chem. Toxicol..

[3] Aprotosoaie A.C., Gille E., Trifan A., Luca V.S., Miron A. (2017). Essential oils of Lavandula genus: A systematic review of their chemistry. Phytochem. Rev..

[4] Khorshidian N., Yousefi M., Khanniri E., Mortazavian A.M. (2018). Potential application of essential oils as antimicrobial preservatives in cheese. Innov. Food Sci. Emerg. Technol..

[5] Bakry A.M., Abbas S., Ali B., Majeed H., Abouelwafa M.Y., Mousa A., Liang L. (2015). Microencapsulation of Oils: A Comprehensive Review of Benefits, Techniques, and Applications. Compr. Rev. Food Sci. Food Saf..

[6] Donsì F., Ferrari G. (2016). Essential oil nanoemulsions as antimicrobial agents in food. J. Biotechnol..

[7] Falleh H., Ben Jemaa M., Neves M.A., Isoda H., Nakajima M., Ksouri R. (2021). Formulation, physicochemical characterization, and anti- E. coli activity of food-grade nanoemulsions incorporating clove, cinnamon, and lavender essential oils. Food Chem..

[8] Huck-Iriart C., Montes-De-Oca-Ávalos J., Herrera M.L., Candal R.J., Pinto-De-Oliveira C.L., Linares-Torriani I. (2016). New insights about flocculation process in sodium caseinate-stabilized emulsions. Food Res. Int..

[9] Sabouri S., Geng J., Corredig M. (2015). Tea polyphenols association to caseinate-stabilized oil–water interfaces. Food Hydrocoll..

[10] Zhao S., Li Y., Liu Q., Xia X., Chen Q., Liu H., Kong B. (2024). Characterization, release profile, and antibacterial properties of oregano essential oil nanoemulsions stabilized by soy protein isolate/tea saponin nanoparticles. Food Hydrocoll..

[11] Li M., Ritzoulis C., Du Q., Liu Y., Ding Y., Liu W., Liu J. (2021). Recent progress on protein-polyphenol complexes: Effect on stability and nutrients delivery of oil-in-water emulsion system. Front. Nutr..

[12] Dickinson E. (2008). Interfacial structure and stability of food emulsions as affected by protein–polysaccharide interactions. Curr. Opin. Colloid Interface Sci..

[13] Anton N., Vandamme T.F. (2010). Nano-emulsions and Micro-emulsions: Clarifications of the Critical Differences. Pharm. Res..

[14] Santos J., Jimenez M., Calero N., Alfaro M., Muñoz J. (2019). Influence of a shear post-treatment on rheological properties, microstructure and physical stability of emulgels formed by rosemary essential oil and a fumed silica. J. Food Eng..

[15] Minakov A., Mikhienkova E., Pryazhnikov M., Voronenkova Y., Skorobogatova A. (2022). Experimental study of the rheological properties and stability of highly-concentrated oil-based emulsions. J. Mol. Liq..

[16] Tian L., Kejing Y., Zhang S., Yi J., Zhu Z., Decker E.A., McClements D.J. (2021). Impact of tea polyphenols on the stability of oil-in-water emulsions coated by whey proteins. Food Chem..

[17] Liu X., Chen L., Kang Y., He D., Yang B., Wu K. (2021). Cinnamon essential oil nanoemulsions by high-pressure homogenization: Formulation, stability, and antimicrobial activity. LWT.

[18] Shlosman K., Rein D.M., Shemesh R., Cohen Y. (2024). Lyophilized Emulsions of Thymol and Eugenol Essential Oils Encapsulated in Cellulose. Polymers.

[19] Lis-Balchin M. (2002). Lavender: The Genus Lavandula.

[20] Dong G., Bai X., Aimila A., Aisa H.A., Maiwulanjiang M. (2020). Study on lavender essential oil chemical compositions by GC-MS and improved pGC. Molecules.

[21] Lin Y., Yu G., Zhang S., Zhu G., Yi F. (2024). Comparative analysis of the differences in volatile organic components of three lavender essential oils in Ili region using sensory evaluation, GC-IMS and GC-MS techniques. J. Chromatogr. A.

[22] Abouelela M.B., El-Taher E.M., Shawky E.M., Baky M.H. (2026). Uncovering adulteration and quality variations in commercial lavender essential oils from the Egyptian market using GC-MS and chemometrics. Sci. Rep..

[23] Meng W., Sun H., Mu T., Garcia-Vaquero M. (2023). Pickering emulsions with chitosan and macroalgal polyphenols stabilized by layer-by-layer electrostatic deposition. Carbohydr. Polym..

[24] Flekka K., Dimaki V.D., Mourelatou E., Avgoustakis K., Lamari F.N., Hatziantoniou S. (2024). Stability and Retention of Nanoemulsion Formulations Incorporating Lavender Essential Oil. Cosmetics.

[25] Li Z.-H., Cai M., Yang K., Sun P.-L. (2019). Kinetic study of d-limonene release from finger citron essential oil loaded nanoemulsions during simulated digestion in vitro. J. Funct. Foods.

[26] Ren X., Yue S., Xiang H., Xie M. (2018). Inclusion complexes of eucalyptus essential oil with β-cyclodextrin: Preparation, characterization and controlled release. J. Porous Mater..

[27] Liao W., Dumas E., Ghnimi S., Elaissari A., Gharsallaoui A. (2021). Effect of emulsifier and droplet size on the antibacterial properties of emulsions and emulsion-based films containing essential oil compounds. J. Food Process. Preserv..

[28] Zheng H., Chen S., Liu Z., Zhou G., Zhang X., Kang S., Hua Q., Wu Y., Liu Z. (2024). Utilization of nanoemulsion to enhance antibacterial capacity of Zanthoxylum bungeanum pericarp essential oils against foodborne pathogenic bacteria. LWT.

[29] Moghimi R., Aliahmadi A., McClements D.J., Rafati H. (2016). Investigations of the effectiveness of nanoemulsions from sage oil as antibacterial agents on some food borne pathogens. Food Sci. Technol..

[30] Vanegas D., Abril-Novillo A., Khachatryan A., Jerves-Andrade L., Penaherrera E., Cuzco N., Wilches I., Calle J., Leon-Tamariz F. (2021). Validation of a method of broth microdilution for the determination of antibacterial activity of essential oils. BMC Res. Notes.

