# Supplementary material for: Sodium Caseinate/Tea Polyphenols Stabilized Lavender Essential Oil Nanoemulsions: Preparation, Characterization, Antibacterial Activity and Potential as Natural Food Preservatives
Source: Polymers (Basel). 2026 Jun 19;18(12):1526. doi: 10.3390/polym18121526 (PMC13306744; doi:10.3390/polym18121526)
Supplement: Supplementary file 1 [file polymers-18-01526-s001.zip › polymers-4251057-supplementary.pdf]

Supplementary Materials

Table S1. Univariate Mean Table.

Factor	level	Particle size (nm)	Zeta-potential (mV)	PDI
A (emulsifier ratio)	1:1	174.63±26.20	-17.61±12.05	0.13±0.01
	1:2	172.63±18.92	-9.90±9.70	0.22±0.06
	2:1	166.2±23.10	-21.69±8.61	0.11±0.02
B (Homogeneous pressure, MPa)	30	190.57±9.14	-16.89±12.48	0.13±0.03
	50	167.20±13.69	-13.37±7.36	0.17±0.10
	70	155.70±20.45	-18.93±13.70	0.17±0.05
C (Homogenization cycle)	3	185.03±13.75	-10.82±6.35	0.15±0.06
	5	167.43±24.71	-11.31±9.87	0.14±002
	7	161.00±19.28	-27.07±5.20	0.17±0.10

Table S2. Apparent Viscosity Data Change.

shear rate(1/s)	Number								
	1	2	3	4	5	6	7	8	9
1.33359	0.0135877	0.0159842	6.42E-03	5.10E-03	2.65E-03	2.69E-03	0.0135877	0.0159842	6.42E-03
1.77835	5.14E-03	5.59E-03	6.87E-03	4.93E-03	1.83E-03	2.23E-03	5.14E-03	6.59E-03	6.47E-03
2.37147	4.21E-03	5.24E-03	5.47E-03	2.59E-03	1.76E-03	9.92E-04	4.21E-03	6.24E-03	5.47E-03
3.1624	2.64E-03	3.01E-03	1.95E-03	2.13E-03	1.57E-03	9.89E-04	2.64E-03	3.01E-03	1.95E-03
4.21714	1.58E-03	1.75E-03	1.10E-03	1.57E-03	1.14E-03	9.88E-04	1.58E-03	1.75E-03	1.10E-03
5.62365	1.71E-03	1.65E-03	1.16E-03	1.51E-03	1.12E-03	9.64E-04	1.51E-03	2.05E-03	1.26E-03
7.49922	1.50E-03	1.58E-03	1.10E-03	1.27E-03	1.11E-03	9.64E-04	1.50E-03	1.58E-03	1.10E-03
10.0004	1.44E-03	1.55E-03	1.05E-03	1.23E-03	1.08E-03	9.63E-04	1.44E-03	1.55E-03	1.05E-03
13.3359	1.38E-03	1.53E-03	8.43E-04	1.19E-03	9.62E-04	9.52E-04	1.38E-03	1.53E-03	8.43E-04
17.7836	1.34E-03	1.50E-03	8.17E-04	1.17E-03	9.30E-04	9.52E-04	1.34E-03	1.50E-03	8.17E-04
23.7147	1.34E-03	1.52E-03	8.24E-04	1.17E-03	9.32E-04	9.47E-04	1.34E-03	1.52E-03	8.24E-04
31.6239	1.30E-03	1.47E-03	8.13E-04	1.13E-03	9.07E-04	9.37E-04	1.30E-03	1.47E-03	8.13E-04
42.1713	1.27E-03	1.45E-03	7.91E-04	1.11E-03	8.88E-04	9.22E-04	1.27E-03	1.45E-03	7.91E-04
56.2366	1.25E-03	1.43E-03	7.78E-04	1.09E-03	8.82E-04	9.10E-04	1.25E-03	1.43E-03	7.78E-04
74.9925	1.24E-03	1.41E-03	7.80E-04	1.08E-03	8.83E-04	9.07E-04	1.24E-03	1.41E-03	7.90E-04
100.005	1.25E-03	1.40E-03	7.07E-04	1.09E-03	9.02E-04	9.04E-04	1.25E-03	1.40E-03	7.87E-04

Table S3. Precipitation of LEO nanoemulsions during storage.

Number	Precipitation			
	Day 1	Day 7	Day 10	Day 20
1	No	No	Mild delamination	Obvious delamination
2	No	No	Mild delamination	Obvious delamination
3	No	No	Mild delamination	Obvious delamination
4	No	Mild delamination	Obvious delamination	Obvious delamination
5	No	Mild delamination	Obvious delamination	Obvious delamination
6	No	Mild delamination	Obvious delamination	Obvious delamination
7	No	No	No	Obvious delamination
8	No	No	No	No
9	No	No	No	Mild delamination

Table S4. Orthogonal design matrix and ANOVA results for particle size, PDI and zeta potential (ANOVA were based on the orthogonal design shown in Table S1.).

**Number**	**A (emulsifier ratio)**	**B (Homogeneous pressure, MPa)**	**C (Homogenization cycle)**	**Particle size (nm)**	**PDI**	**Zeta-potential (mV)**
1	1:1	30	3	200.8	0.1268	-16.06
2	1:1	50	5	174.7	0.1289	-6.411
3	1:1	70	7	148.4	0.1426	-30.37
4	1:2	30	5	187.7	0.1619	-4.849
5	1:2	50	7	151.4	0.2831	-21.08
6	1:2	70	3	178.8	0.2238	-3.756
7	2:1	30	7	183.2	0.09213	-29.77
8	2:1	50	3	175.5	0.1033	-12.63
9	2:1	70	5	139.9	0.1295	-22.67
	Factor	Degrees of freedom	Sum of squared deviations	Mean square value	F	Significance
Particle size (nm)	A (emulsifier ratio)	2	116.51	58.25	0.35	0.74
	B ((Homogeneous pressure, MPa))	2	1893.94	946.97	5.68	0.15
	C (Homogenization cycle)	2	928.75	464.37	2.79	0.26
	Error	2	333.27	166.63		
	Total	8	3272.46			
Zeta-potential (mV)	A (emulsifier ratio)	2	215.32	107.66	3.23	0.24
	B ((Homogeneous pressure, MPa))	2	47.44	23.72	0.71	0.58
	C (Homogenization cycle)	2	513.05	256.52	7.69	0.12
	Error	2	66.73	33.37		
	Total	8	842.54			
PDI	A (emulsifier ratio)	2	2.19E-02	1.09E-02	7.12	0.12
	B ((Homogeneous pressure, MPa))	2	3.52E-03	1.76E-03	1.15	0.47
	C (Homogenization cycle)	2	1.64E-03	8.18E-04	0.53	0.65
	Error	2	3.07E-03	1.54E-03		
	Total	8	0.03			
